# Retrospective assessment of a collaborative digital asthma program for Medicaid-enrolled children in southwest Detroit: reductions in short-acting beta-agonist (SABA) medication use

**DOI:** 10.1186/s40733-023-00092-0

**Published:** 2023-05-20

**Authors:** Meredith Barrett, Rahul Gondalia, Vy Vuong, Leanne Kaye, Alex B. Hill, Elliott Attisha, Teresa Holtrop

**Affiliations:** 1 ResMed Science Center, San Diego, USA; 2 IQVIA; Formerly ResMed Science Center, Durham, USA; 3 Department of Urban Studies and Planning, Wayne State University; formerly Detroit Health Department, Detroit, USA; 4 Formerly Detroit Public Schools Community District, Detroit, USA; 5 Wayne Children’s Healthcare Access Program, Inc, Dba Kids’ Health Connections, Detroit, USA

**Keywords:** Asthma, Short-acting beta-agonist, SABA, Digital health, Electronic monitoring, Sensor, Medicaid, Pediatric

## Abstract

**Background:**

Real-world evidence for digitally-supported asthma programs among Medicaid-enrolled children remains limited. Using data from a collaborative quality improvement program, we evaluated the impact of a digital intervention on asthma inhaler use among children in southwest Detroit.

**Methods:**

Children (6–13 years) enrolled with Kids Health Connection (KHC), a program involving home visits with an asthma educator, were invited to participate in a digital self-management asthma program (Propeller Health). Patients were provided with a sensor to capture short-acting beta-agonist (SABA) medication use, and given access to a paired mobile app to track usage. Patients’ healthcare providers and caregivers (“followers”) were invited to view data as well. Retrospective paired t-tests assessed change in mean SABA use and SABA-free days (SFD) over time, and regressions explored the relationship between followers and medication use.

**Results:**

Fifty-one patients were assessed. Mean program participation was nine months, and patients had on average 3 followers. From the first to last participation month, mean SABA use decreased from 0.68 to 0.25 puffs/day (*p* < 0.001), and mean SFD increased from 25.2 to 28.1 days/month (*p* < 0.001). 76% of patients had an increase in the number of SFD. There was a positive, but non-significant, relationship between the number of followers and reductions in SABA inhaler use.

**Conclusions:**

We observed a significant reduction in SABA inhaler use and an increase in the number of SABA-free days among Medicaid-enrolled children enrolled in a multi-modal digital asthma program.

## Background

Detroit, Michigan wasonce described as an "epicenter of asthma burden" and in 2022 was named the foremost Asthma Capital [1, 2], with high prevalence among children and adults. Nationally, asthma is estimated to impact 6% of children and 8% of adults [3], whereas in Detroit, prevalence estimates are approximately doubled, with 15% of children and 16% of adults estimated to be living with asthma [4].

Disparities also exist with prevalence differences by age, race and gender, as well as geography, such that individuals residing in low-income communities are at increased risk for developing asthma, and experience higher rates of asthma-related healthcare utilization claims compared to their peers. In Detroit, children with asthma visit the emergency department at about double the rate of their counterparts in the state of Michigan, and Black adults and children are more likely to have an asthma-related hospitalization than White adults and children with asthma [4]. Further, given the association between pollution and asthma outcomes, concerns about air quality in the Detroit region have persisted for decades with particular emphasis on the heavily industrialized southwest area of the city, where a large proportion of underserved households reside [5].

Digital interventions for asthma have been associated with improved outcomes including improved disease control, medication adherence, and reduced short-acting beta-agonist (SABA) medication use [6–8]. Further, compared to traditional, non-digital interventions, digital interventions provide an opportunity to enhance condition management via improved patient and provider communications, tailored education materials, passive monitoring of symptoms and medication taking behaviors, and possible reductions in healthcare disparities [9, 10].

However, real-world implementation and evidence of digital health programs among Medicaid-enrolled populations remain limited and lack of access may further contribute to the digital divide, which has been well documented in the United States [11, 12]. Investigating the impact of digital health interventions in asthma is especially important within at-risk groups, such as children of lower socioeconomic status, to help limit the disparities in asthma morbidity.

Therefore, Kids’ Health Connections (KHC), Detroit Public Schools Community District (DPSCD) and the Detroit Health Department partnered with a commercially-available digital self-management asthma platform to deliver a collaborative, digitally-supported asthma quality improvement program among Medicaid-enrolled children in southwest Detroit. The program aimed to evaluate changes in asthma outcomes, including SABA medication use and the frequency of symptom-free days among enrolled patients.

## Methods

### Collaborative Model

#### Kids’ Health Connections (KHC) [13]

KHC is a not-for-profit organization based in Detroit, Michigan, which supports patients, families, and healthcare providers through education and healthcare services. Children with asthma are typically referred to KHC following an emergency department visit, or through their healthcare provider, to receive more holistic asthma care and education regarding medication management, inhaler technique and trigger awareness. Upon referral, an asthma educator is assigned to the child and their family to support the child’s goals as outlined in their asthma action plan. Asthma educators conduct 4–6 home visits with families, a school visit with the patient and staff, and a companion visit with the patient’s healthcare provider to share learnings.

#### Detroit Public Schools Community District (DPSCD)

DPSCD has a vested interest in children with asthma, as asthma-related absenteeism is associated with poorer educational outcomes compared to classmates without asthma-related absenteeism [14]. Through a public–private collaboration with KHC, DPSCD helps to promote KHC’s asthma services through school nurses, and school and healthcare events.

#### Digital platform for asthma management

**Fig. 1:**
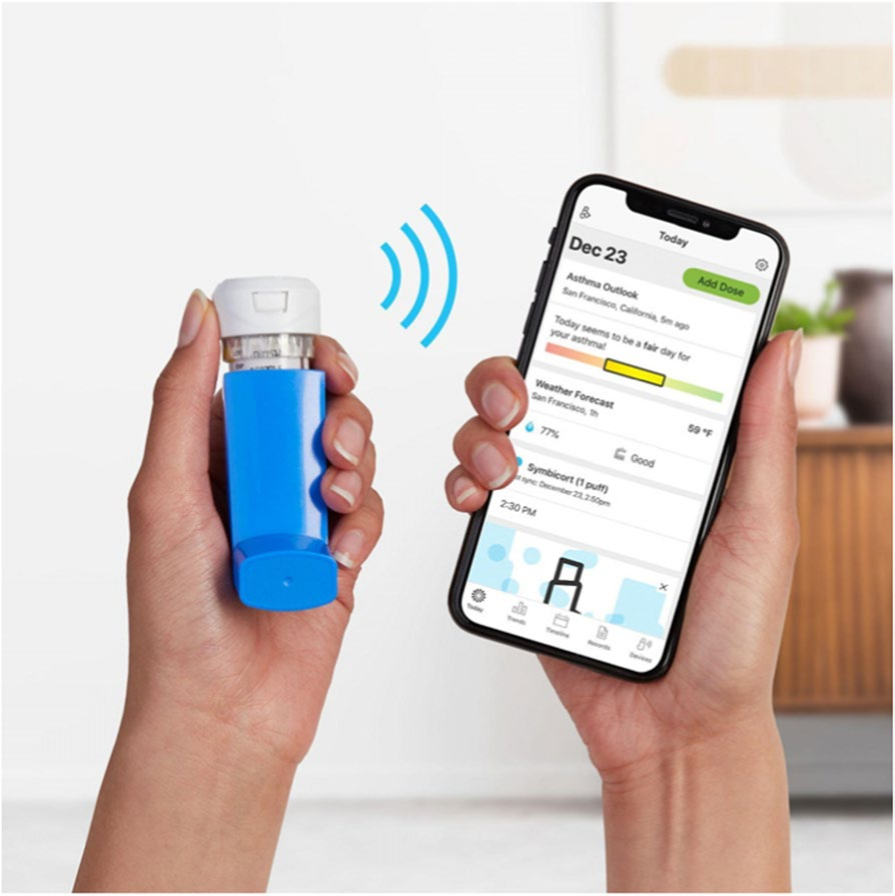
The digital self-management platform included a medication sensor to capture inhaler use and a companion smartphone app to receive medication reminders and education

In an effort to further support the relationship between KHC asthma educators, patients and providers, patients and their caregivers were invited to enroll with a digital self-management platform (Propeller Health, Wisconsin) [7, 8], which included an inhaler medication sensor that attached to the patient’s short-acting beta-agonist (SABA) inhaler (Fig. 1). The sensor captured the time and date of each inhaler actuation. This data was then transmitted wirelessly via Bluetooth to a patient-facing app on the patient’s smartphone. The app provided education and feedback on inhaler usage. Patients also received sensor and app-based medication reminders to use daily controller inhalers, and were prompted by the app to complete the Asthma Control Test (ACT) during enrollment and monthly thereafter. Platform content was delivered in either English or Spanish. Caregivers and primary care providers (PCPs) were invited to monitor patient data in the app and web-based clinician portal, respectively, and discuss trends with the patient’s KHC asthma educator.

### Patient recruitment and program design

Children (6–13 years of age) with self-reported asthma living in four ZIP codes of southwest Detroit were eligible to participate in the program. Patients were recruited by KHC and DPSCD through in-clinic enrollment, schools, health fairs, and local community events from December 2018 to December 2019. Eligible patients who agreed to participate in the digitally-enhanced KHC asthma education program enrolled in the digital platform during the first home visit with the asthma educator. The KHC asthma educator helped patients place medication sensors on the patient’s compatible rescue and controller inhalers, and then synced the sensor(s) with the paired smartphone app to allow for data to be transferred from the sensor to the software platform. Patients (or their caregiver, if applicable) were not required to have a smartphone in order to participate. Families without a smartphone may have synced their sensor via a portable hub that plugged in at home or school and synced with the patients’ sensors. Access to inhaler medication use data was granted to eligible healthcare providers, including asthma educators and PCPs, as well as patient caregivers if desired. KHC educators would outreach to PCPs as necessary to discuss a patient’s medication use trends based on available objective data, but only PCPs would make treatment adjustments. Data on the frequency of patient outreach was not available for this study.

### Study analyses

This study is a retrospective analysis of data collected as part of the quality improvement program described above. Patients with 60 or more days of sensor data were included. Daily mean SABA use, defined as mean daily puffs per person, and SABA-free days (SFD), defined as a full calendar day in which no SABA use was captured, were assessed throughout the program. Paired t-tests (ɑ = 0.05) estimated change in mean daily SABA use and SFD from first to last month of participation. The number and percentage of patients who had at least one more SFD (“improved”), the same amount of SFDs (“maintained”), and at least one SFD fewer (“worsened”) in the last (vs. first) month of monitoring was also examined. Asthma control was determined by averaging patient responses to the monthly ACT or Childhood ACT (cACT), with uncontrolled asthma defined as ACT ≤ 19 [15, 16].

Exploratory analyses examined the association between the number of patient followers (defined as the number of patient-specific caregivers, healthcare providers, and KHC asthma educators following a unique patient) and SABA usage (mean puffs/day and SABA-free days). These analyses were restricted to patients who had any SABA use recorded during the study period. Linear regressions were used for these analyses but did not include any confounding variables due to limited data availability.

A *p*-value < 0.05 (2-tailed) was considered to be statistically significant in this study.

All users agreed to Propeller’s Terms of Use, and retrospective analyses were determined by the WCG Institutional Review Board to be exempt from further review.

## Results

Data from 51 patients was assessed: mean (SD) patient age was 11.5 (2.4) years, and 29.4% had uncontrolled asthma at baseline. Mean (SD) program retention was 269.1 (171.75) days (Table 1). Most patients (68.6%) had at least two followers added to their account.

**Table 1:** Patient characteristics

Characteristic	*N* = 51
**Age (mean (SD)), years**	11.5 (2.4)
**Gender, n (%)**	
*Female*	12 (23.5)
*Missing*	10 (19.6)
**Asthma control at enrollment, (mean (SD))**	
*Uncontrolled (ACT* ≤ *19), n (%)*	15 (29.4)
*Controlled (ACT* > *19), n (%)*	20 (39.2)
*Missing, n (%)*	16 (31.4)
**Days in program (per SABA syncing)**	290.4 (185.5)
**Month 1 (baseline) SABA usage data**	
*Daily SABA use (mean (SD)), puffs/day*	0.68 (1.11)
*Daily SABA use (median(IQR)), puffs/day*	0.27 (0.13, 0.98)
*Weekly SABA use (mean (SD)), puffs/week*	4.79 (7.77)
*Weekly SABA use (median(IQR)), puffs/week*	1.87 (0.93, 6.88)
*SABA-free days (mean (SD)), days*	25.2 (4.8)
*SABA-free days (median(IQR)), days*	27 (22.5, 29)
**Number of SABA medications on hand (patient-reported)**	
*1*	32 (62.7)
*2*	18 (35.3)
*3*	1 (2.0)
**Number of followers per patient, mean (SD)**	3.20 (2.2)
**Patients with ≥ 2 followers, n (%)**	35 (68.6)
Number of patients with a compatible controller inhaler	9 (17.6)

### Change in SABA use

From the first to last month, mean SABA use decreased from 0.68 to 0.25 puffs/day (-0.43, 95% CI: -0.67, -0.20; *p* < 0.001), and mean SFD increased from 25.2 to 28.1 days/month (2.9 days, 95% CI: 1.7, 4.1, *p* < 0.001) (Table 2). Over the study period, 76% of patients had an increase in the number of SFD, 7.8% had no change in the number of SFD, and 15.7% of patients had fewer SFD (Table 3).

**Table 2:** SABA-free days in the last vs. first month of Propeller, among users with at least two full months of Propeller usage (*n* = 51)

	**First month**	**Last month**	**Change**	**Mean (95% CI)**	***P* value**
SABA puffs/day	0.68	0.29	Absolute (puffs/day)	-0.43 (-0.67, -0.20)	*p* < 0.001
SABA puffs/week	4.79	2.03	Absolute (puffs/week)	-2.76 (-4.6, -0.96)	*p* = 0.003
SABA-free days	25.2	27.8	Absolute (days)	2.6 (1.2, 4.0)	*p* = 0.0004
			Relative (%)	12.5 (5.7, 19.4)	*p* = 0.0006

**Table 3:** Number (%) patients who improved, maintained or worsened SABA-free days from first to last month of monitoring (*n* = 51)

Change over time	N (%)
Improved	40 (78.4)
Maintained	3 ( 5.9)
Worsened	8 (15.7)

### Exploratory followers analysis

Each patient had a mean (SD) of 3.2 (2.2) followers, with a maximum of 6 (Table 1). Although not statistically significant, there was a positive association between the number of followers and SABA-free days, with a stronger relationship observed with an increasing number of followers (Table 4), such that a patient with two to three followers had on average 3% more SABA-free days compared to a patient with one follower. A similar, non-significant relationship was observed for SABA use (Table 4), such that a patient with more than three followers had on average 0.44 puffs/day less than a patient who had one follower.

**Table 4:** Linear regression models estimating the association between the number of followers and SABA use among Propeller users with over 1 day of use (*n* = 59)

	**Number of Followers**	**N** **(Number of Patients)**	**Estimate**	**Std. Error**	**P**
**SABA-free days**	1 (reference)^a^	21	–	–	–
	2–3	19	3%	5%	0.62
	> 3	19	9%	5%	0.09
**SABA Use (mean puffs/day)**	**1 (reference)**^**a**^	**21**	**–**	**–**	**–**
	2–3	19	-0.02	0.25	0.94
	> 3	19	-0.44	0.25	0.08

LInear regression assumptions were checked with residual plots and histograms and were deemed satisfactory

^a^Those with 1 follower, the reference group, all had a caregiver as their follower. Those with over 1 follower had one caregiver and at least one physician follower

## Discussion

In this retrospective analysis of a collaborative, clinically-integrated digital program, we observed improved clinical outcomes among Medicaid-enrolled children with asthma living in southwest Detroit. Specifically, we observed a significant reduction in SABA inhaler use, and a mean increase of almost three additional SABA-free days, with over 75% of patients experiencing an improvement in the number of days without needing to use their SABA inhaler.

This quality improvement program highlights the strength of a collaborative, comprehensive model and further supports the importance of hybrid approaches [17] to support promising outcomes in difficult to reach patients with asthma residing in the United States. Of note, there was a relatively high number of followers per patient within this population. While not statistically significant, the positive association between the number of followers and SABA usage merits further study in a larger sample.

This program also suggests that facilitating communications across patient followers, including healthcare providers, asthma educators, and caregivers, and with patients themselves, may support better asthma outcomes, especially when paired with patient self-management tools and digital insights on medication usage. Indeed, past studies have demonstrated that digital insights that are shared not only with a patient, but also with their healthcare provider, resulted in additional improvements in asthma outcomes [18, 19]. However, more work is needed to better understand the mechanisms of improvement, the type of follower involvement necessary (e.g., provider vs. caregiver vs. asthma educator), and the frequency of follower interaction needed to promote improved asthma outcomes [6, 20].

Given the small, single cohort analyzed in this quality improvement program over a limited duration, the observed results should be considered in light of its design limitations. Future studies should confirm findings in a larger sample and consider a comparison group over a longer period of time to help mitigate some of these design issues.

Further, about one-third of patients had missing baseline asthma control data, limiting the ability to fully characterize the enrolled sample. While improvements in SABA use are observed in other randomized controlled trials [4, 6], it is possible the results observed in this program may be the result of regression to the mean and/or the Hawthorne effect [21, 22]. In other words, the change in SABA use may be a reflection of the natural variation of SABA use over time, or the change may be due to the participant’s perception of increased oversight (due to enrollment in the digital platform which provided data to the patient and/or their caregiver, as well the patient’s healthcare provider). Both of these limitations could be addressed through difference in difference analyses or propensity score matching, but require a larger sample for meaningful output. It is also possible that patients did not add a sensor to all of their inhaler medications, especially in patients who may have resided in multiple households, thus limiting the ability to capture all inhaler usage. Similarly, very few patients had a compatible sensor for their controller inhaler, limiting insights on medication adherence and disease management. Finally, because not all patients participated in a full year of the program, it is possible that seasonality may have played a role, with summer inhaler usage typically observed to be lower than in winter months [23].

Despite these limitations, this program highlights the promise of a hybrid approach to asthma management, marrying clinical and digital tools to enhance transparency of care for the patient, their caregivers and their provider. This program was also supported through public and private partnerships, which can hold promise when it comes to funding and providing resources. Continued sustainability of such a collaboration may be sought through policy changes to support reimbursement for remote therapeutic monitoring, as well as continued public–private partnerships in respiratory care.

## Conclusions

We observed a statistically significant reduction in SABA inhaler use and an increase in the number of SABA-free days among Medicaid-enrolled children who were enrolled in a multi-modal, collaborative digital asthma program in southwest Detroit. Future studies should confirm findings in a larger, prospective study, and plan to measure the impact of specific program components.

## Data Availability

Upon reasonable request, the de-identified datasets used to support the conclusions of this article may be made available.

## Abbreviations

ACT: Asthma Control Test
DPSCD: Detroit Public Schools Community District
KHC: Kids’ Health Connections
PCP: Primary care provider
SABA: Short-acting beta-agonist
SD: Standard Deviation
